# Therapeutic efficacy of sulphadoxine-pyrimethamine and chloroquine for the treatment of uncomplicated malaria in pregnancy in Burkina Faso

**DOI:** 10.1186/1475-2875-5-49

**Published:** 2006-06-15

**Authors:** Sheick Oumar Coulibaly, Désiré Nezien, Salifou Traoré, Bibiane Koné, Pascal Magnussen

**Affiliations:** 1 Laboratoire National de Santé Publique, 09 BP 24 Ouagadougou 09, Burkina Faso; 2 Centre Medical Paul VI, 01 BP 2099 Ouagadougou 01, Burkina Faso; 3 UFR Sciences de la Santé, Université de Ouagadougou, 03 B.P. 7021 Ouagadougou 03, Burkina Faso; 4 DBL – Institute for Health Research and Development, Copenhagen, Jaegersborg Allé 1 D DK-2920 Charlottenlund, Denmark

## Abstract

**Background:**

A reduction in the therapeutic efficacy of chloroquine (CQ) and sulphadoxine-pyrimethamine (SP) has recently been observed in Burkina Faso. As these two drugs are used in pregnancy, their efficacy in pregnant women was studied to directly assess the level of drug resistance in this specific population, rather than to extrapolate results of studies conducted in children < 5 years of age.

**Methods:**

During the malaria transmission season of 2003 in Ouagadougou, the clinical efficacy of SP and CQ, using the WHO 28-day protocol, was assessed in primigravidae and secundigravidae presenting with uncomplicated malaria.

**Results:**

PCR-corrected results by day 28 showed that among 62 women treated with SP, eight (12.9%) experienced late parasitological failure, but no clinical failures. Among 60 women treated with CQ, the overall failure rate was 46.7% including 1.7% early treatment failures, 5% late clinical failures and 40% late parasitological failures. SP induced a haemoglobin gain of 0.3 g/dL by day 14 and 0.9 g/dL by day 28. Treatment responses were independent of gravidity, gestational age and prior antenatal care visits.

**Conclusion:**

While CQ should no longer be used, the efficacy of SP is still compatible with use for intermittent preventive treatment (IPT) in pregnancy. However, given the possible spread of resistance, the drug should be restricted in its use.

## Background

Malaria remains one of the major infectious diseases threatening the health of pregnant women in Africa [1]. Pregnancy associated malaria affects birth outcome by causing maternal anaemia, stillbirths, abortion and low birth weight infants [2-9].

Case management of malaria in pregnancy should include effective and safe drugs to ensure maternal recovery and prevent adverse consequences for the foetus. When implementing new treatment and prevention policies, particular interest should be given to pregnant women as the number of antimalarial drugs safe to be used in pregnancy is limited.

It is only recently that Burkina Faso, where malaria is endemic but with marked seasonal transmission, has experienced increasing levels of resistance to chloroquine (CQ) and to some extent to sulphadoxine-pyrimethamine (SP) [10-14]. Historically, CQ has been used as the first-line drug for treatment of the general population, including pregnant women, and SP was used as second-line treatment. CQ was used for weekly chemoprophylaxis in pregnancy. A new malaria treatment policy was formulated in February 2005, whereby CQ was replaced by arthemether/lumefantrine (Coartem) as first-line drug for uncomplicated malaria, while intermittent preventive treatment (IPT) with SP was introduced to replace CQ prophylaxis in pregnancy. SP has, however, rarely been used for case management in pregnancy and, therefore, its efficacy in treating malaria in pregnant women is not known.

The aim of this study was to provide information on the efficacy of both CQ and SP in pregnant women with uncomplicated malaria. The study also aimed at providing a better understanding of the relationship between the levels of resistance found in children and in pregnant women.

## Methods

### Study site

The study was conducted between September and December 2003 at the 'Centre Médical avec Antenne Chirurgicale Paul VI' of Ouagadougou, the capital city of Burkina Faso. This health centre is situated in the north-western peri-urban part of the city and is the referral hospital for the corresponding health district. It comprises a maternity ward with Antenatal Care (ANC) services where pregnant women can attend at affordable costs.

Ouagadougou is located within the Sudano-Sahelian zone, with a tropical climate characterized by two seasons: a long dry season (November – May) and a rainy season lasting from June to October. In 2003, the total rainfall was 848 cm and the average monthly temperature ranged from 25°C to 33°C (National Meteorological Service, Ouagadougou, 2005). Malaria is endemic with a markedly seasonal transmission (July to November) [15,16]. *Plasmodium falciparum *is the most common parasite species. Until recently, CQ was used as the first-line drug for clinical malaria management and for chemoprophylaxis in pregnancy (at a weekly dose of 300 mg) whilst SP was the second-line drug. However, SP was rarely used in pregnancy and not for IPT. More than 80% of pregnant women attend ANC at least once (general population census, 1995). Bed nets are not commonly used by pregnant women (Wedraogo et al, unpublished). The prevalence of HIV infection among adults in 2003 was 4.2% (95% CI: 2.7% – 6.5%) (UNAIDS, 2004). In 2002, a sentinel site surveillance found an HIV infection rate of 4% among pregnant women [17].

### Study protocol

The study was conducted as a clinical efficacy trial following the protocol proposed by the WHO for monitoring antimalarial drug resistance (WHO/CDS/CSR/EPH/2002.17- WHO/CDS/RBM/2002.39) [18]. A 28-day follow-up design was used. The main outcomes assessed were treatment efficacy and impact on haemoglobin levels.

The inclusion criteria were as follows:

- Primigravidae or secundigravidae with gestational age between 12 and 36 weeks (fundal height less than 30 cm);

- Axillary temperature ≥ 37.5°C, recent history of fever (within the 48 hours preceding enrolment) or presence of clinical malaria-related symptoms ;

- mono-infection with *P. falciparum *with a density ≥ 2000 parasites/mm^3 ^of blood;

- Absence of clinical sign of severe malaria [19];

- Absence of other patent infections;

- Absence of previous severe reaction to CQ or SP;

- Staying in a neighbouring district or village;

- Able to come for follow-up.

- Consent

The exclusion criteria were the following:

- Clinical signs of severe malaria [19];

- Other febrile disease than malaria;

- Use of interfering treatment during the follow-up period;

- High-risk pregnancy.

Thick blood smears were prepared at Days 0, 2, 3, 7, 14, 21 and 28. Blood collection on filter papers was also done at the time of making the slides. Haemoglobin (Hb) measurement was done on days D0, D14 and D28. Axillary body temperature, at inclusion and at each follow up visit, was measured using an electronic thermometer.

The interpretation and classification of treatment outcome was performed according to the WHO protocol for low to moderate transmission areas [18]. The classification was as follows: adequate clinical and parasitological response (ACPR), early treatment failure (ETF), late clinical failure (LCF) or late parasitological failure (LPF). In cases of treatment failures occurring on D14, D21 or D28, merozoite surface protein 1 and 2 (*msp1 *and *msp2*) PCR genotyping was used to distinguish recrudescent parasitaemia from re-infection [20].

### Patient recruitment, treatment and follow up

Primigravidae and secundigravidae attending the outpatient department (OPD) or the ANC services and fulfilling the inclusion criteria were screened for malaria parasites. Those with a positive blood slide were invited to participate in the trial. Oral informed consent was obtained from subjects before inclusion. Personal demographic information, medical and obstetrical history and a detailed history of the current illness were recorded, and a clinical and obstetrical examination performed. Gestational age was estimated by eliciting information on the date of the woman's ultimate menstrual period combined with the fundal height.

The women were randomly assigned to CQ or SP using a computer generated random number list. The study participants were seen daily for the following 3 days for control of temperature, clinical history, clinical examination, and for those assigned to the CQ group, treatment. Thereafter, they were seen on days 7, 14, 21, 28 for evaluation. SP and CQ were procured from UNICEF, Copenhagen. CQ tablets (100 mg) were administered over 3 days as: 10 mg/kg/day on days 0 and 1, and 5 mg/kg/day on day 2. SP tablets were administered as a single dose of 25 mg sulphadoxine/kg + 1.25 mg pyrimethamine/kg. In practice, a quarter of tablet was given per 5 kg body weight. Treatment administration was directly observed, and patients were observed for the subsequent thirty minutes. In case of treatment failure, oral quinine (25 mg/kg bodyweight/day in 3 doses) over five days was given as treatment. Women who had already attended ANC and who were supposed to be under CQ chemoprophylaxis were asked to stop chemoprophylaxis but to continue with iron and folic acid supplementation.

Women were invited to present as soon as possible to the research team if any complication arose during the follow-up period. At each follow up visit, a clinical history was taken, women were clinically examined if necessary, temperature was measured and blood obtained by finger prick. The ones who did not return for scheduled follow up visits at the clinic were visited at home. All information on clinical history, clinical examination, parasitological and haematological examinations were recorded in pre-designed and tested forms and continuously updated.

### Laboratory methods

Finger prick blood was used to prepare thick and thin blood films (D0 and follow up days), for Hb measurement (D0, D14 and D28), and for blood spot sampling on filter paper (*Whatmann 3 MM *chromatography filter paper cut into strips of 0.5 × 3 cm) for PCR (D0 and follow up days). The thick films were air-dried, stained with Giemsa, and read using an oil immersion lens at 100× magnification. Asexual parasites were counted against 200 leucocytes and the parasite density per mm^3 ^of blood was estimated assuming a value of 8,000 leucocytes/mm^3 ^of blood. At least 100 thick film fields were examined before a slide was considered negative. The parasite species were determined on the thin film. All slides were examined by two different microscopists. When the readings differed, the slide was read by a third microscopist, whose results were retained as definitive.

Haemoglobin levels were measured using a portable battery powered photometer (*HemoCue*^® ^AB, Ängelholm, Sweden). Anaemia was defined as Hb < 11 g/dL. An Hb level between 8 and 11 g/dL was considered mild to moderate anaemia and an Hb level ≤ 8 g/dL as severe anaemia.

Polymerase Chain Reaction (PCR) assays were performed on blood collected on filter papers. Genotyping for merozoite surface protein 1 and 2 (*msp1 *and *msp2*) genes was performed to distinguish recrudescence of the original parasite strains from reinfection with new parasite strains as described by Snounou & Beck [20]. Parasite DNA was extracted with methanol. The polymorphic regions of *msp-1*, and *msp-2*, were amplified by nested PCR [21]. Nested PCR products were analyzed by electrophoresis using 1.5% agarose gel. Genotype patterns were compared for each allele between parasites of D0 and DR (parasite recrudescence day). An outcome was defined as recrudescence if the DR sample contained identical alleles or a subset of the alleles present in the D0 sample and as re-infection if the DR sample contained alleles not present in the D0 infection.

### Statistical analysis

The data from the case record forms were double entered using Epi- info 6.04 fr (CDC, Atlanta, USA). Data were analysed using Epi-info and SPSS 13. Continuous normally distributed data were described by the mean, standard deviation, and non-normally distributed data by the median or geometrical mean and range. Data from women lost to follow up were excluded from analysis. Proportions were compared using the Chi square test and normally distributed continuous variables were compared using Student's t test.

### Ethical considerations

The study was approved by the National Ethical Review Committee in Burkina Faso, authorized by the Ministry of Health and recommended by the Danish National Committee on Biomedical Research Ethics. An oral informed consent was obtained from all the women before participation in the study.

## Results

### General characteristics of pregnant women recruited for the trial

**Figure 1:**
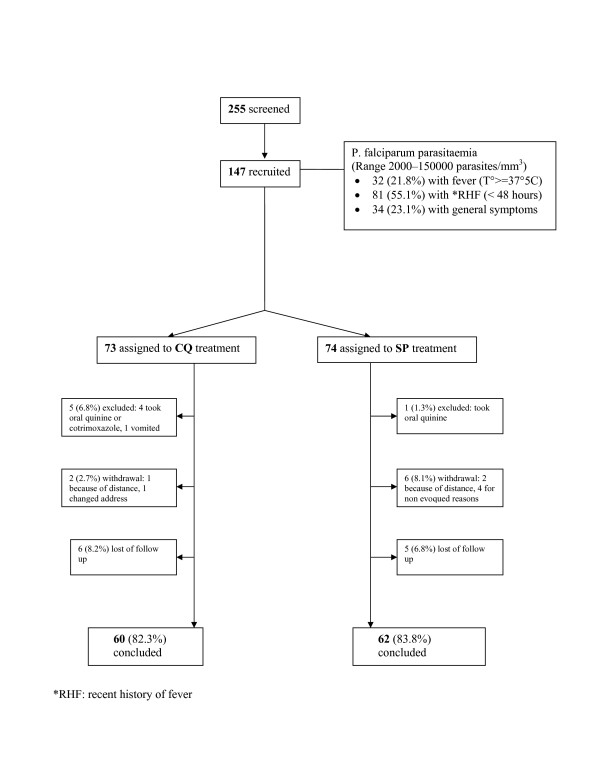
Trial profile. (*RHF: recent history of fever)

Among 943 primigravidae and secundigravidae who consulted ANC or OPD between September 21^st ^and December 8^th ^2003, 255 were in the second or third trimester of pregnancy and presented with symptoms and signs leading to a presumptive diagnosis of acute and uncomplicated malaria and were screened for malaria infection. One hundred and two women were excluded as they were either aparasitaemic or had parasitaemia < 2000 parasites/μL whilst one was infected with *P. malariae*. A total of 147 women (68% primigravidae and 32% secundigravidae) fulfilling all inclusion criteria were finally recruited (Figure 1). Median age was 20 years (range 15 to 29 years). Based on fundal height measurements, 107 (72.8%) of the women were categorized as being in the second trimester of pregnancy, and 40 (27.2%) in the third trimester. Ninety four (63.9%) of them had not attended ANC during their pregnancy. Seventy-seven (52.4%) had no formal education, most being housewives (79.6%) without any income generation of their own. Ninety-nine (81%) of the women lived in less developed areas of the district. Seventy three (49.7%) women said they owned a bed net.

The reasons for consulting the OPD were mainly: a recent history of fever (55.1%), fever on the day of presentation (21.8%) and unspecific symptoms (21.1%). These included: headache, fatigue, muscle aches and general body pains. Forty women presented with fever (axillary temperature >= 37.5°C) with a mean temperature of 37.9°C (SD ± 0.4). Mean Parasitaemia at recruitment was 7768 asexual parasites/mm^3 ^[range 6353–9498] and mean Hb was 9.4 g/dl (SD ± 1.5).

Seventy three (49.7%) and 74 (50.3%) were assigned to the CQ and the SP group, respectively. Among them, 16 (13.1%) had sought care at a health centre more than a month before recruitment. Presumptive malaria had been diagnosed in nine cases. Five had been treated with CQ and four had been treated with oral quinine.

Among the 147 enrolled women, six were excluded because they had taken other drugs during the follow-up period, eight withdrew from the study for various reasons and 11 were lost to follow up. One hundred and twenty two completed the study with 60 in the CQ group and 62 in the SP group, respectively (Figure 1). Baseline characteristics of the women who completed the 28-day follow-up are summarized in Table 1. No adverse reaction attributable to treatment were reported to the research team neither those who completed the follow up or nor from those who withdrew by themselves.

**Table 1:** Baseline characteristics of pregnant women with uncomplicated malaria who completed the 28 days therapeutic efficacy assessment in Ouagadougou, 2003.

Baseline characteristics	Total (n = 122)	Treatment group	
		
		CQ (n = 60)	SP (n = 62)
Median age in years [range]	20 [15 – 29]	20 [15 – 28]	19.5 [15 – 29]
Primigravidae	83 (68%)	41 (68.3%)	42 (67.7%)
Secundigravidae	39 (32%)	19 (31.7%)	20 (32.3%)
Presented with fever	28 (22.9%)	19 (31.7%)	9 (14.5%)
Temperature (mean)	37.9 (± 0.5)	38 (± 0.5)	37.8 (± 0.3)
Parasite count (geometric mean/mm^3^) [range]	7874 [6269–9889]	8055 [5840–11111]	7699 [5523–10732]
Haemoglobin D0 (g/dL)	9.4 (± 1.6)	9.5 (± 1.6)	9.3 (± 1.6)
Anaemia:			
- severe (Hb<8 g/dL)	23 (18.8%)	12 (20%)	14 (22.6%)
- mild to moderate (Hb 8-<11 g/dL)	73 (59.9%)	38 (63.3%)	35 (56.4%)
- Absence (11+ g/dL)	26 (21.3%)	10 (16.7%)	13 (21%)

### Parasiteamia and gametocyteamia

At inclusion, parasiteamia ranged from 2000 to 150000 parasites/mm^3 ^with a median of 6000 parasites/mm^3^. Only two patients had a parasiteamia ≥ 100,000 parasites/mm^3^. There was no significant difference in mean parasiteamia between primigravidae (7922 [5941–10564]) and secundigravidae (7768 [5302–11380]) *P *= 0.937. The prevalence of gametocytes on the day of recruitment was 4%. It remained around 4% in the two treatment groups apart from D7 where an increase was observed in both CQ group (6.7%) and SP group (17.7%) and among the CQ group at D28 (8.8%). No gametocytes were observed at D28 among the eight women in the SP group with LPF. Gametocytes were associated with recrudescence of asexual parasites in 19.4% of the failures.

### Anaemia

The prevalence of anaemia among the women was 81% (21% with severe anaemia and 60% with mild to moderate anaemia). At admission there was no significant difference in mean Hb between primigravidae (9.3 g/dL ± 1.6) and secundigravidae (9.5 g/dL ± 1.6, *P *= 0.65). Curiously, mean Hb, at inclusion, was higher in women who had not previously attended ANC (9.6 g/dL ± 1.6) compared with those who had attended at least once (9.0 g/dL ± 1.4, *P *= 0.04) and who were supposed to have received chemoprophylaxis with CQ and iron/folic acid supplementation.

In patients successfully treated with CQ, no significant gain on Hb level was evident at D14 (*P *= 0.49) nor at D28 (*P *= 0.34) as compared to D0. On the other hand, patients treated with SP gained 0.38 g/dL at D14 (*P *= 0.006) and 0.9 g/dL at D28 (p < 10^-3^). Although mean Hb levels were found to be lower at D14 or D28 among treatment failures, the reduction was not significant.

### Therapeutic efficacy

The PCR corrected results on therapeutic efficacy at day 14 and day 28 are presented in Table 2. One case of LPF in the CQ group was reclassified as a re-infection by PCR. Almost half of the women (28, 46.7%) treated with CQ were not cured of their infection. Most demonstrated LPF. In the SP group failure (all LPF) occurred in 12.9%.

**Table 2:** CQ and SP therapeutic assessment cumulative results at day 14 and day 28 in pregnant women in Ouagadougou, 2003.

Assessment results	CQ treatment group (n = 60)		SP treatment group (n = 62)	
	
	D14	D28	D14	D28
ETF	1 (1.7%)	1 (1.7%)	0 (0%)	0 (0%)
LCF	1 (1.7%)	3 (5%)	0 (0%)	0 (0%)
LPF	6 (10%)	24 (40%)	3 (4.8%)	8 (12.9%)
ACPR	52 (86.6%)	32 (53.3%)	59 (95.2%)	54 (87.1%)
Total	60(100%)	60(100%)	62(100%)	62(100%)

ETF: early treatment failure

LCF: late clinical failure

LPF: late parasitological failure

ACPR: adequate clinical and parasitological response

There were as many treatment failures in primigravidae as in secundigravidae with CQ (43.9% and 47.4%, respectively (*P *= 0.8)) and SP (14.2% and 10%, respectively (*P *= 0.6)). Failures rates were also similar irrespective of the trimester of pregnancy (*P *= 0.77). The frequency of treatment failures was not different between women who had attended ANC at least once (30.4%) and those who had no prior attendance to ANC (27.3%, *P *= 0.74).

Treatment failures were found in three of the nine women who had reported a recent illness episode diagnosed as presumptive malaria. All three of them had received oral quinine for treatment. Recrudescent parasiteamia at D14 (CQ: 5, SP: 1) or at D28 (CQ: 7, SP: 6) were usually of low density, ranging from 40 to 2,000 parasites/mm^3^.

According to the modified classification of parasitological response [22], 13.3% early RI, 36.6% late RI, and 1.6% RII were found in the CQ treatment group while 1.6% early RI, 9.7% late RI, and 3.2% RII were found in the SP treatment group. No case of RIII response was found to any treatment group.

## Discussion

In the present study the efficacy of CQ and SP was assessed in primigravidae and secundigravidae presenting with uncomplicated malaria. CQ and SP had until recently been recommended in Burkina Faso as antimalarial drugs for first and second line treatment. The study was undertaken to specifically assess the treatment efficacy among pregnant women as they may be considered a distinct subgroup of the population, with regard to malaria immunity and treatment outcomes. Usually antimalarial drug efficacy has been assessed in non-immune children less than 5 years of age, but no data are available on the correlation between antimalarial drug efficacy assessed in children under five years of age and efficacy in other segments of the population. Although antimalarial immunity is impaired in pregnancy, especially among primigravidae, the level of immunity is considered to be higher in adult pregnant women than among under-five year olds [23-25] and a high level of treatment failures among this young age group might not be reflected in pregnant women. In Burkina Faso, anti-malarial prophylaxis with CQ given in sub-therapeutic weekly dose regimens is available for pregnant women attending ANC. This might select for resistant strains, allow asymptomatic parasite carriage [26,27] and be the explanation for anaemia and treatment failures. It is, therefore, of public health interest to assess the drugs efficacy in such a specific group. Furthermore it was recently found that CQ chemoprophylaxis was not effective [11] due to high levels of parasite resistance. In this context, it was found it useful to assess the efficacy of SP among pregnant women before introducing this drug for IPT as replacement for CQ prophylaxis.

The standardized WHO protocol for low to moderate transmission areas was adapted for use with pregnant women in whom clinical malaria doesn't always present with typical symptoms. Malarial fever may not occur in adults even in pregnant women with some degree of impaired immunity [28,29]. In this study, consideration was mainly given to anamnestic signs and history of fever associated with parasite carriage in order to increase the number of subjects enrolled and to take into account those women who presented without fever but who complained of malaria related symptoms in association with parasites in peripheral blood. Thus, only 21.8% of enrolled women presented with fever on the day of enrolment. Unfortunately the follow-up did not include specific inquiries about the disappearance of the symptoms present at inclusion but rather assessed the general clinical improvement which, when associated with parasite clearance, could be considered as a recovery. Safety was not specifically addressed in this study, but both SP and CQ are commonly used during pregnancy and known to be safe when administered at the recommended doses: CQ throughout the pregnancy and SP in the second and third trimester [30].

In the CQ group, ETF were observed in only 1.7% of cases while LCF were observed in 5% of cases, bringing total clinical failures to 6.7%. The incidence of clinical failures was low in this study and would have been acceptable if not associated with a high incidence of LPF (40%) which indicates that when extending follow-up beyond 14 days the level of resistance to CQ makes it unsuitable for treatment. On the other hand, no case of clinical failure, either ETF or LCF was found in the SP group, all failures being LPF (12.9%). These findings indicates that SP has retained a reasonable level of efficacy for treatment of uncomplicated malaria in pregnancy and that it still remains a valuable drug for IPT. However, there is a serious risk that resistance to SP may develop quickly if used for general malaria case management further compounded by the widespread use of cotrimoxazole, another sulpha drug which could generate cross resistance with SP [31,32].

A 28-day efficacy study was conducted in under-five year old children at the same period as the present study in a neighbouring health district of Ouagadougou [10]. Overall failure rates of 63.4% (87/137) to CQ and 13.8% (17/125) to SP were recorded. ETF rates were higher within the CQ group than in the SP group. These failure rates were higher than the 46.7% observed in pregnant women for CQ (*P *= 0.02, RR= 1.36 CI95%: 1.01–1.83), but similar to the 12.9% observed for SP (*P *= 0.89). Furthermore, ETF was more common in children than in pregnant women. These observations suggest that, beyond a general decrease in CQ and SP efficacy, failure rates to CQ are lower in pregnant women than in children. This is probably due to the higher level of humoral immunity in pregnant women [23]. However, efficacy rates remained comparable for SP under the same conditions.

Contrary to CQ, SP induced a gain in mean Hb by day 14 which further increased by day 28, indicating that malaria is an important contributing factor to anaemia in pregnancy [33]. Women attending ANC are supposed to take chemoprophylaxis and iron and folic supplementation to prevent anaemia. Testing CQ in women under chemoprophylaxis could be seen as unsuitable due to the long half life of CQ, but no data were available regarding the compliance with the chemoprophylactic regimen. Furthermore, it was common practice at the health facilities to treat malaria attacks in pregnant women with CQ and the efficacy of CQ and SP was not found related to ANC attendance. In Burkina Faso regular attendance to ANC services is low. Most of the women included in the study came for treatment of an illness rather than for attending ANC although the visit at the OPD was used by the health centre staff to attempt to enrol the women for ANC. Complaints about side effects of CQ, especially bitterness and drowsiness are common, and although compliance was not assessed in this study, it might be very low. This could be the explanation of a lower mean Hb level on the day of inclusion in women who had at least visited ANC once prior to the admission visit.

## Conclusion

CQ has lost its efficacy for malaria case management in pregnant women in the study area and chemoprophylaxis with this drug cannot effectively prevent malaria in pregnancy [11]. Its use in pregnancy should be replaced with alternative drugs and this was recently implemented by the Ministry of Health of Burkina Faso which changed to a policy of IPT with SP given once in the second and third trimester (Ministry of Health, February 2005). With ETF less than 15%, SP must still be considered as being reasonably efficacious and, in the absence of alternatives, its use for case management remains possible. However, since widespread use of SP can rapidly lead to resistance [34-37] it's use for only IPT in pregnant women rather than for treatment is recommend.

## Authors' contributions

SOC conceived the study and its design, he participated and coordinated the collection of data. He analysed the data and drafted the manuscript. PM helped in designing the study, interpreting the results and critically revised the manuscript. BK contributed in designing the study and revising the manuscript. DN and ST participated in coordination of data collection and data analysis. All authors read and approved the final manuscript.
